# Comparative Analysis of Audio Processing Techniques on Doppler Radar Signature of Human Walking Motion Using CNN Models

**DOI:** 10.3390/s23218743

**Published:** 2023-10-26

**Authors:** Minh-Khue Ha, Thien-Luan Phan, Duc Hoang Ha Nguyen, Nguyen Hoang Quan, Ngoc-Quan Ha-Phan, Congo Tak Shing Ching, Nguyen Van Hieu

**Affiliations:** 1 Department of Physics and Electronic Engineering, University of Science, Vietnam National University of Ho Chi Minh City, Ho Chi Minh City 70000, Vietnam; hmkhue@hcmus.edu.vn (M.-K.H.); ptluan@hcmus.edu.vn (T.-L.P.); nhquan@hcmus.edu.vn (N.H.Q.); 2 Graduate Institute of Biomedical Engineering, National Chung Hsing University, Taichung 402, Taiwan; 3 Faculty of Information Technology, University of Science, Vietnam National University of Ho Chi Minh City, Ho Chi Minh City 70000, Vietnam; ndhha@fit.hcmus.edu.vn; 4 Independent Researcher, Ho Chi Minh City 70000, Vietnam; hpnq.work@outlook.com

**Keywords:** doppler radar, motion classification, deep learning

## Abstract

Artificial intelligence (AI) radar technology offers several advantages over other technologies, including low cost, privacy assurance, high accuracy, and environmental resilience. One challenge faced by AI radar technology is the high cost of equipment and the lack of radar datasets for deep-learning model training. Moreover, conventional radar signal processing methods have the obstacles of poor resolution or complex computation. Therefore, this paper discusses an innovative approach in the integration of radar technology and machine learning for effective surveillance systems that can surpass the aforementioned limitations. This approach is detailed into three steps: signal acquisition, signal processing, and feature-based classification. A hardware prototype of the signal acquisition circuitry was designed for a Continuous Wave (CW) K-24 GHz frequency band radar sensor. The collected radar motion data was categorized into non-human motion, human walking, and human walking without arm swing. Three signal processing techniques, namely short-time Fourier transform (STFT), mel spectrogram, and mel frequency cepstral coefficients (MFCCs), were employed. The latter two are typically used for audio processing, but in this study, they were proposed to obtain micro-Doppler spectrograms for all motion data. The obtained micro-Doppler spectrograms were then fed to a simplified 2D convolutional neural networks (CNNs) architecture for feature extraction and classification. Additionally, artificial neural networks (ANNs) and 1D CNN models were implemented for comparative analysis on various aspects. The experimental results demonstrated that the 2D CNN model trained on the MFCC feature outperformed the other two methods. The accuracy rate of the object classification models trained on micro-Doppler features was 97.93%, indicating the effectiveness of the proposed approach.

## 1. Introduction

Various methods and technologies have been utilized for environmental monitoring in modern times, including video cameras [1], infrared sensors [2], ultrasonic sensors [3], and radar sensors [4]. However, each method has its limitations. For example, video surveillance technology has issues with ensuring user privacy [5], and intrusion alarm systems employing ultrasonic and infrared sensors often generate false alarms [6]. Recent studies have therefore focused on combining radar technology with machine learning to develop effective surveillance systems that can overcome these limitations. The emergence of AI radar technology [7,8] offers several advantages over other technologies, including low cost, privacy assurance, high accuracy, and environmental resilience, among others. Despite these potential benefits, the use of AI radar technology is limited due to high equipment costs and a lack of radar datasets required for training deep-learning models. The scarcity of recently proposed radar training datasets represents a significant barrier to the widespread adoption of AI radar technology in our daily lives [9]. Moreover, traditional radar signal processing techniques encounter challenges such as inadequate resolution. Despite efforts to address this issue through the adoption of methods like continuous wavelet transform (CWT) [10] and the S-method [11], which promise precision, practical applications have revealed their inefficiency due to their complex computational requirements.

Micro-Doppler radar signals generated by moving objects provide rich information about their motion patterns. Changes in the time–frequency characteristics of these signals can be leveraged to recognize the micro-motions of the target [12]. A Doppler radar is capable of transmitting an electromagnetic (EM) wave with a specific wavelength, denoted as λ. The signal emitted from the radar can be mathematically represented as:

(1) $$\begin{array}{c} x_{t}\left(t\right)=A_{t}\cos\left(2\pi f_{0}t+\phi \left(t\right)\right) \end{array}$$

where $f_{0}$ and $\phi \left(t\right)$ represent the carrier frequency and phase. The reflected signal from the radar can be expressed as:

(2) $$\begin{array}{c} \begin{array}{c} x_{r}\left(t\right)=A_{r}\cos\left(2\pi (f_{0}+f_{D})t+\phi \left(t\right)\right) \end{array} \end{array}$$

By utilizing the Doppler effect, the velocity of the moving object can be determined. The frequency shift at the center, known as the Doppler frequency, can be expressed as:

(3) $$\begin{array}{c} f_{D}=\frac{2v}{\lambda }cos\theta \end{array}$$

In this context, v is the velocity of the moving object, and the incident angle to the radar is denoted by θ. For Doppler radar signal processing, the high-frequency carrier signal is filtered out, retaining only the baseband signal containing the Doppler signatures. The baseband signal arises from the mixing of the transmitted and received signals and can be expressed as follows:

(4) $$\begin{array}{c} \begin{array}{c} x_{D}\left(t\right)=\mathrm{Acos}\left(2\pi f_{D}t+\phi \left(t\right)\right) \end{array} \end{array}$$

In the presence of complex-motion objects, comprising M nonstationary parts exhibiting accelerated radial velocities, distinct frequency shifts are induced in the incident signal due to the Doppler effect. These motions give rise to additional Doppler shifts known as micro-Doppler effects. In this scenario, the resulting baseband signal, containing the micro-Doppler effect, can be modeled as a combination of M sine-wave signals, represented as follows:

(5) $$\begin{array}{c} \begin{array}{c} x_{D}\left(t\right)=\overset{M}{\underset{i=1}{\sum }}A_{i}\cos\left(2\pi f_{Di}t+\phi_{i}\left(t\right)\right) \end{array} \end{array}$$

Thus, a motion can be represented as a sequence of micro-Doppler signatures of echo signals. A comprehensive examination of the relationship between human locomotion and micro-Doppler characteristics is outlined in [13,14,15,16]. Recent approaches, including a theoretical model [17] and experimental results [18,19,20], have been explored to prove the difference in the micro-Doppler signature between humans and other moving objects such as animals or vehicles. Therefore, to maintain a more concise approach, the primary focus of this study is the characterization of the micro-Doppler signature associated with human activities. Two main classes of human movement, walking and walking without arm swing, were chosen for the method’s provability.

**Figure 1:**
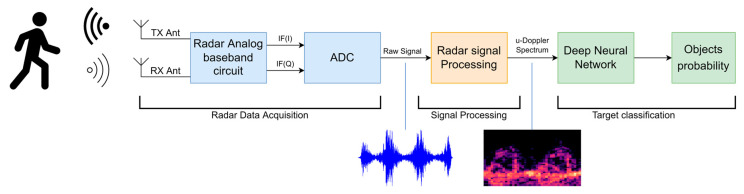
Block diagram overview of the main parts of the Doppler radar system.

In recent years, machine learning has risen as a powerful classification tool that utilizes algorithms to automatically identify patterns and make predictions based on data. By analyzing large amounts of labeled or unlabeled data, machine learning algorithms can learn and adapt, enabling accurate classification of new or unseen instances with high efficiency. Many studies on motion classification using deep learning based on the micro-Doppler signature have been conducted in various contexts and applications, such as hand-gesture classification [21], cough detection [7], and pedestrian detection [22,23]. In common with most related studies, a Doppler radar system typically comprises the main blocks illustrated in Figure 1 of the system block diagram. In this system, two notable advancements have emerged in the study of micro-Doppler signature analysis. Firstly, deep neural networks (DNNs), and specifically, convolutional neural networks (CNNs) have been utilized as both feature extractors and classifiers. Secondly, the time–frequency representation of raw radar signals, known as spectrograms, have been used as inputs.

Studies have been conducted throughout the last decades to explore automatic classification methods for human motion using a Doppler radar. Most techniques extract micro-Doppler features from time, frequency, joint time–frequency (T–F), or joint time–scale (T–S) domains. The short-time Fourier transform (STFT) is a frequently employed method for micro-Doppler signature analysis, as noted in [9]. However, its time and frequency resolutions are limited by the fixed window length, resulting in a spectrogram with many lumps due to inadequate time–frequency analysis resolution. Other methods, including the continuous wavelet transform (CWT) [10] and the S-method [11], have been considered for micro-Doppler signature analysis. Nevertheless, these methods have not been found to be effective in practical implementation due to their computational complexity. The time–frequency representation method used in this study draws inspiration from effective audio processing algorithms such as mel spectrogram and MFCCs (mel-frequency cepstral coefficients) due to their analogy with Doppler radar and sound signals. They have been shown to be powerful methods with the computational efficiency to visually represent audio signals as inputs to convolutional neural networks (CNNs) [24,25]. While the mel spectrogram and STFT are often used to analyze the frequency content of a signal over time, MFCCs are used to capture the shape of the spectral envelope of the signal. The mel spectrogram and STFT are both sensitive to noise and can produce inaccurate results in the presence of noise. On the other hand, MFCCs are less sensitive to noise and can be more robust in noisy environments. However, the choice of feature extraction technique depends on the specific application and the characteristics of the signal being analyzed. In some cases, mel spectrogram or STFT may be more appropriate, while in other cases, MFCCs may be more suitable. In terms of micro-Doppler signature analysis, it is important to evaluate the performance of each technique for the specific application and choose the one that provides the best results. Despite the common and conventional STFT, the employment of MFCCs in representing doppler signature from a radar has been done in the automotive industry [26], fall detection applications [27], or respiring rate analysis [28], which proves its effectiveness and flexibility in many applications. In addition to the feature extraction techniques mentioned earlier, the classification models utilized also play a crucial role in achieving accurate classification. In the field of deep learning, convolutional neural networks (CNNs) have been widely employed for representation learning of visual imagery. Deep CNNs have contributed significantly to the progress in image understanding in recent times. In the domain of speech recognition systems, Abdel-Hamid et al. [29] pioneered the use of CNN-based models for phone recognition. Furthermore, studies such as [30,31] have indicated that CNNs have been successful in the task of human motion classification as well.

Recently, there have been many studies in human motion classification utilizing the benefits from radar technology and artificial intelligence [11,32,33,34]. However, these studies mostly contributed to the classification models and their signal processing approaches, STFT and the S-method, but have not analyzed robustness or efficiency. Therefore, in this study, the research group proposed a novel signal processing technique for analyzing micro-Doppler signatures associated with human motion. The group then employed various machine-learning models to classify different types of human motion based on the processed micro-Doppler data. A custom-design hardware prototype was used to acquire radar motion data in the K band frequency—24 GHz. Three different signal processing techniques, namely STFT, mel spectrogram, and MFCCs, were employed to obtain micro-Doppler spectrograms for each motion category: non-human motion, human walking, and human walking without arm swing. To classify the micro-Doppler signatures, a simplified 2D CNN architecture was utilized, treating the micro-Doppler spectrogram as a 2D feature representation. Additionally, ANN and 1D CNN models were implemented for comparative analysis in terms of accuracy, computational complexity, and model size. The primary aim of our comparison was to assist future researchers in making informed decisions about algorithm selection, parameter tuning, and robustness assessment. By doing so, the research group hopes to contribute to the development of more reliable and effective methods for micro-Doppler signature analysis. These advancements have broad applications, including in healthcare, security, and automotive systems, making our study valuable for various real-world scenarios.

## 2. Materials and Methods

### 2.1. Radar Data Acquisition System

**Figure 2:**
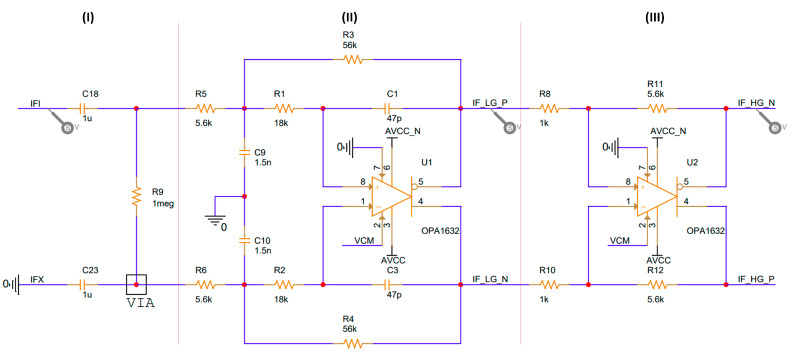
Analog baseband circuit with fully differential MFB topology. (**I**) DC removal, (**II**) low-pass filter and amplifier, and (**III**) auxiliary amplifier.

A K-Band Transceiver IPS-354 (InnoSent, Donnersdorf, Germany) was used as a continuous wave radar sensor operating in the 24 GHz-ISM-Band. The sensor features a split transmit and receive antenna; however, the amplitude of the analog IF output signal can be quite low (ranging from μV to mV) depending on the distance and radar cross section (RCS) of the target. To amplify these low-amplitude signals, analog amplifiers were used, and a simple DC block circuit with a pair of RC was employed to address the unsteady common mode voltage (DC component) of the Doppler signal. The frequency characteristics of the signal were also analyzed to avoid the aliasing effect during the analog-to-digital conversion stage, and the analog baseband circuit must condition the ADC block by controlling the output signal’s DC level to meet the input voltage range of the ADC. To fulfill these requirements, the differential multiple-feedback (MFB) filter topology was employed (Figure 2), which featured two stages of low-noise analog baseband amplifiers with a bandpass characteristic. The OPA1632 fully differential operational amplifier was used to achieve high-performance amplification.

**Figure 3:**
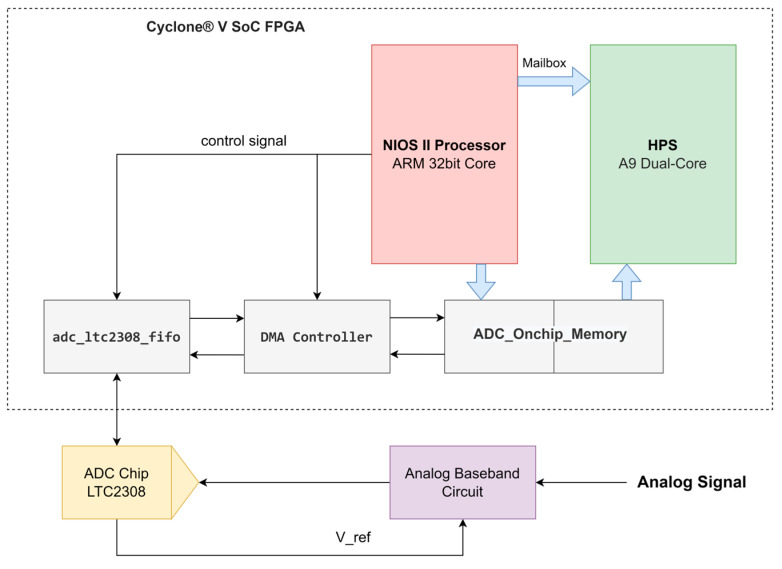
Block diagram of the data acquisition system built on a DE10-Nano Kit.

The hardware platform used for the radar data acquisition circuitry was a Terasic DE10-Nano Kit (Terasic Inc., Hsinchu County, Taiwan), which integrates the Intel System-on-Chip (SoC) FPGA along with a dual-core Cortex-A9 embedded core and a programmable logic gate array (Figure 3). To convert analog Doppler signals to digital data for storage and processing, the onboard LTC2308 chip with a 12-bit resolution and a maximum sampling frequency of 500 kSPS was utilized. This IC met the requirements for 24 GHz Doppler radar signals.

The data acquisition phase comprises two distinct tasks: (1) controlling the operation of the ADC and (2) storing the raw data to flash memory. Task (1) is managed entirely on the Nios processor, which is specifically designed from the FPGA to regulate the flow of ADC data from the ADC_LTC2308_FIFIO module to the shared memory between the Nios core (low-end processor) and the ARM A9 core (high-level processor). On the other hand, task (2) is executed on a Linux operating system operating on an ARM A9 processor core, where the transformed ADC data stored in the shared data memory is read in segments.

### 2.2. Signal Processing

**Figure 4:**
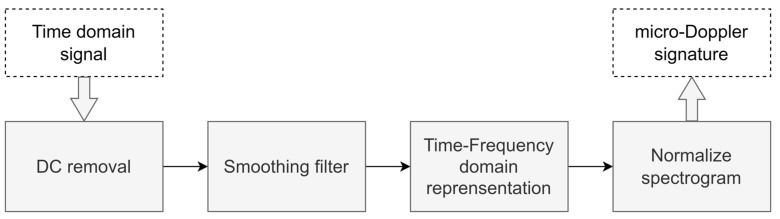
Flow diagram of the entire digital signal processing process.

Signals in the time domain do not provide sufficient information about the object compared to those in the time–frequency domain, which significantly impacts the quality of the signal characteristics. As a result, preprocessing steps are required to eliminate redundant components and reduce interfering signal components in the time domain signal. Python language tools are used to execute the entire signal processing, and a flowchart depicting the processing steps is displayed in Figure 4. Firstly, the DC component in the raw signal in the time domain is eliminated as it does not provide useful information about the moving object. The remaining AC component of the signal is then smoothed using a Butterworth lowpass filter.

The conversion of the signal to the time–frequency domain is a crucial processing step that facilitates the extraction of the micro-Doppler spectrum, which is essential for the signal’s characteristic quality and the classifier’s accuracy. Therefore, signal transformation was conducted using various methods to enable evaluation and comparison. Three primary transformation techniques were deployed, namely the short-time Fourier transform (STFT), mel spectrogram, and mel-frequency cepstral coefficients (MFCCs) [35]. STFT is a widely used time–frequency analysis technique employed to examine the spectral properties of a signal within short time segments. By applying the Fourier transform to overlapping sections of the signal, it yields a time-varying representation of the signal’s frequency components. This enables the investigation of how the signal’s spectral content evolves over time. The mel spectrogram, on the other hand, is a spectrogram representation of an audio signal in which the frequency scale is transformed into the mel scale. The mel scale is designed to mimic the human perception of sound, capturing the characteristics of how different frequencies are perceived. By converting the frequency scale to the mel scale, the mel spectrogram provides valuable insights into the distribution of frequency content over time, aiding in the analysis of the signal’s spectral characteristics. MFCCs are a commonly used feature-extraction technique in the field of speech and audio processing. They capture the spectral envelope of a signal by first taking the logarithm of the magnitudes of the mel spectrogram, then applying the discrete cosine transform (DCT), and finally selecting a subset of coefficients. MFCCs have proven to be highly effective in representing the essential characteristics of the human voice. As a result, they find wide application in tasks such as speech recognition, speaker identification, and music genre classification.

All three methods were implemented using the same processing window configuration with the support of Librosa [36], which is a Python package for music and audio analysis. A window length of 1024 points and a Fourier transform length of 1024 points were chosen. To maintain a balance between spectrogram resolution and computational processing volume, a hop length of 512 (equivalent to 50% overlap length) was selected. In the case of the MFCC method, a total of 128 MFCCs were computed and extracted. The type-2 DCT was chosen to transform the mel spectrogram magnitudes into the cepstral domain. This specific choice of parameters ensured an effective representation of the spectral envelope of the micro-Doppler signature. Furthermore, the resulting spectrogram was normalized to retain only the fundamental features that were crucial for characterizing the micro-Doppler signature. This normalization process helped enhance the discriminative properties of the features, enabling more accurate classification and analysis of the motion patterns.

### 2.3. Classification Model

After processing, the micro-Doppler features were then input into a neural network for classification. To investigate the signal processing methods and analyze their performance under different models, three common models were selected: ANN, 1D CNN, and 2D CNN. The following section provides a comprehensive description of these models, offering detailed insights into their designs.

The architecture of the ANN model consists of layers of interconnected neurons, typically used for tabular or unstructured data such as text. In this study, the ANN model was designed with 7 dense layers (or fully connected layers) of varying widths. The model includes a total of 1,410,803 trainable weights. The first dense layer receives the input feature data, and subsequent dense layers learn these features. The primary part of the model comprises the first 6 dense layers, each equipped with a ReLU activation function.

The second model used was the 1D CNN, whose architecture employs 1D convolutional layers with filters to extract patterns along a single dimension. The input data features are reshaped into a dimension vector (128 × 1). The data passes through a series of layers, starting with a 1D convolutional layer. After the first convolutional layer, a batch normalization layer is applied to expedite network learning convergence. Following that is a max-pooling layer with a pooling window length of 3, reducing computational complexity. In tiers 2, 3, and 4, the BatchNorm layer is replaced by the Dropout layer, randomly dropping nodes with a probability of 0.3. The latter part of the model includes a Dense layer used to synthesize features into a feature vector of length 1024, followed by a Softmax layer to compute probabilities.

The applied 2D CNN model is designed for classifying micro-Doppler feature data in a compact manner, viewing it as 2D data. The model was kept simple, featuring only two convolution layers. Each layer used a 3 × 3 convolution kernel with a Tanh activation function. The output passes through a 2-dimensional max-pooling layer with a size of 2 × 2, combined with a 0.1 probability Dropout layer. The resulting features were flattened using a Flatten layer and synthesized in a Dense layer with a Tanh activation function to produce the final classification result, determined by a Dense layer with a Softmax activation function.

In summary, the choice between ANN, 1D CNN, and 2D CNN depends on the nature of the data and the specific task at hand. ANNs are more general-purpose but may not perform as well on structured data like images or sequences. One-dimensional CNNs are tailored for sequential data analysis, while 2D CNNs excel in image-related tasks by considering both spatial dimensions.

## 3. Experimental Design

**Figure 5:**
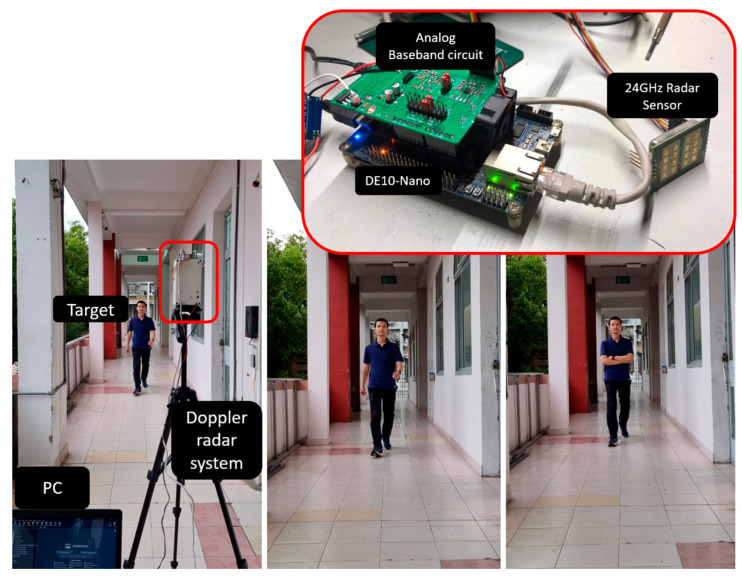
Experimental installation of the radar data-acquisition system.

The dataset comprised data collected from six individuals, of whom five were male and one was female, aged between 22 and 25 years, with a height range of 1.5 m to 1.75 m. Each subject performed two types of movements: normal walking and walking without swinging their arms (Figure 5). The radar sensor continuously received signals while the subjects moved in both directions, towards and away from the radar. The entire dataset, consisting of 4100 samples across 71 min, was randomly divided into three separate sets for training, testing, and validation. There were slight differences in data amounts between the classes. “Human walking without arms swinging” had the most data, with 27 min. “Human normal walking” had 25 min of data, and the “non-human” class had 19 min. The partitioning ratio was set at 72%, 20%, and 8%, respectively.

**Figure 6:**
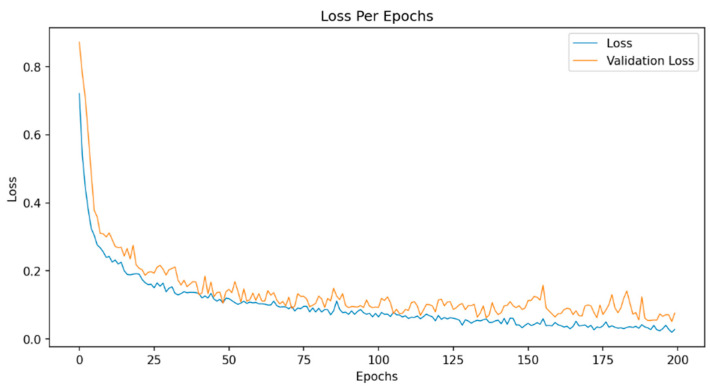
Training process with the 2D CNN model on the MFCC feature.

The training process for each model involved 200 epochs, with a batch size of 32 samples. The Adam optimization algorithm was utilized to optimize the learning rate value, with an initial learning rate of 1 × 10^−4^. Details of the model training parameters are presented in Table 1. To evaluate the training process, the 2D CNN model trained on the MFCC feature was selected as the optimal model to analyze the variations in loss during the training epochs (Figure 6).

## 4. Results and Discussion

### 4.1. Developed Doppler Radar System Performance

**Figure 7:**
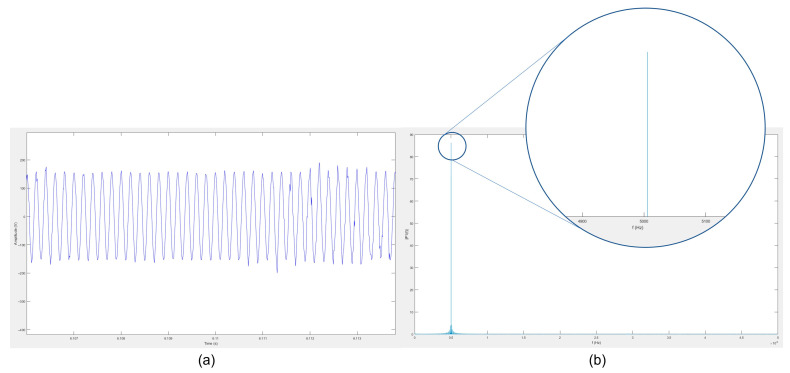
Raw Doppler signals acquired using the designed system depicted in (**a**) the time domain and (**b**) the frequency domain. The signals were obtained using a 5 kHz sine input wave.

In order to evaluate the performance of the implemented hardware system, a reference sine wave with a frequency of 5 kHz was introduced to the analog baseband circuit, as shown in Figure 7a. The system’s sampling rate was set to 48,000 samples/second, ensuring compliance with the Nyquist frequency requirement for capturing the Doppler signal. As illustrated in Figure 7b, the peak frequency of the collected signal was close to the value of 5 kHz. The observed frequency variance was within an acceptable range and did not significantly impact the subsequent processing results, owing to the limited range of the Doppler frequency shift.

### 4.2. Comparative Analysis of T–F Representation Methods

**Figure 8:**
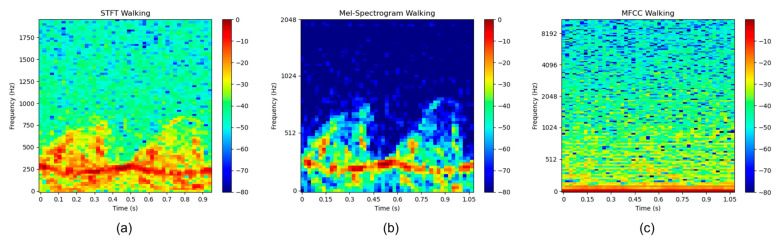
Micro-Doppler spectrogram of human walking in three time–frequency representation methods: (**a**) STFT, (**b**) Mel-spectrogram, and (**c**) MFCC.

When examining the micro-Doppler signature of human walking obtained using the STFT method (Figure 8a) and the mel-spectrogram method (Figure 8b), it became evident that the spectrogram exhibited distinct signature patterns indicative of a walking human, as compared to the simulation results described in [17]. On the other hand, when analyzing the micro-Doppler signature generated by the MFCC method (Figure 8c), it became challenging to determine the presence of human motion in the spectrogram by visual inspection alone. However, contrary to intuitive evaluation, the CNN models perceived these features in a significantly different manner.

**Figure 9:**
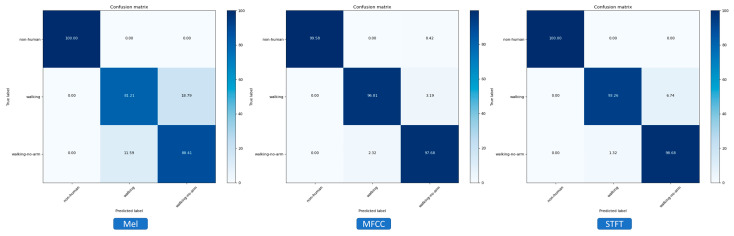
Confusion matrix comparison analysis of 2D CNN model structures trained on three different features.

In order to evaluate and compare the performance of different methods, three primary models were selected: artificial neural network (ANN), one-dimensional convolutional neural network (1D CNN), and two-dimensional convolutional neural network (2D CNN). In this section, the focus is on the comparative analysis of micro-Doppler signature representation methods. Therefore, only the results obtained from the best-performing classification model are discussed, which was the 2D CNN. A confusion matrix was utilized to visualize the model’s prediction accuracy on each feature class (Figure 9). The classifier’s performance can easily be assessed by comparing the diagonal elements with the remaining ones. Upon examining the confusion matrix, it can be observed that the 2D CNN model classification results had uniform accuracy for MFCC and STFT features. However, for the 2D CNN model trained on the mel-spectrogram feature, the confusion rate between the two classes of walking objects with and without arm movement was quite significant. In general, all methods of time–frequency representation effectively captured the distinctions between different classes of walking motion.

**Figure 10:**
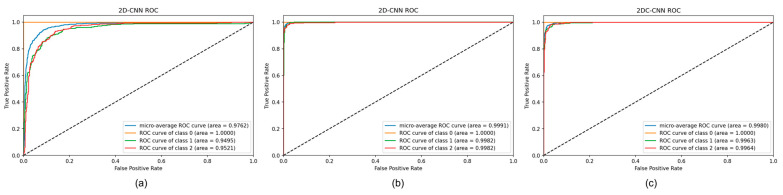
ROC diagram and AUC analysis of models trained on (**a**) mel-spectrogram, (**b**) MFCC, and (**c**) STFT features.

In addition to confusion matrix analysis, the receiver operating characteristic (ROC) and area under the curve (AUC) were used to demonstrate the effectiveness of the methods employed. ROC analysis is a powerful tool for evaluating and comparing the performance of classification models. It plots the true positive rate (TPR) against the false positive rate (FPR) at different classification thresholds. AUC is a common metric used to quantify the overall performance of a classifier. Based on the ROC diagram presented in Figure 10, it can be observed that the model trained on the MFCC feature (Figure 10b) outperformed the models trained on the mel-spectrogram (Figure 10a) and STFT (Figure 10c) features, with an AUC value close to 1. Additionally, the difference in ROC among the three classes was relatively small, indicating a balanced classification rate of the model across all classes.

**Figure 11:**
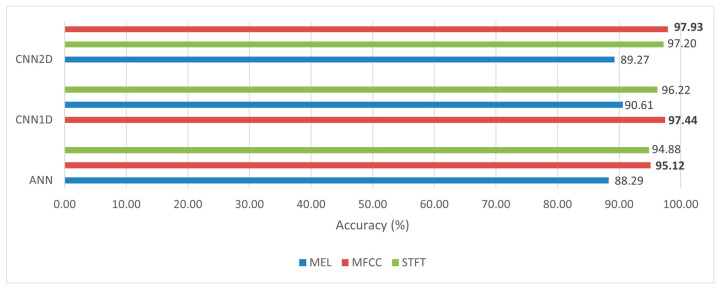
Comparative analysis of model accuracy on different T–F representation methods. Remarks: bold number represent the highest accuracy achieved.

Numerically, the best prediction results were obtained from the 2D CNN model trained on MFCC data, with an accuracy of 97.93% (Figure 11). Following this, the 2D CNN model trained on the STFT feature achieved an accuracy of 97.2%, and finally, the 2D CNN model trained on the mel-spectrogram feature obtained an accuracy of 89.27%. Representing the spectrogram in the mel scale inadvertently discarded certain fine-grained frequency features associated with motion, resulting in a loss of generality. Consequently, models using the mel-spectrogram feature exhibited a significantly lower prediction rate compared to that of other models. Based on the numerical and graphical evaluation methods, it was evident that the MFCC method yielded superior results compared to those of the STFT and mel-spectrogram methods. This can be attributed to the fact that the MFCC features eliminated the global frequency range variation caused by changes in human walking speed, thanks to the frequency envelop characteristics. As a result, the learned feature was more general and less susceptible to specific object-related influences. The 2D CNN model trained on the MFCC feature achieved the highest accuracy of 97.93%, while the 1D CNN model trained on the mel-spectrogram feature performed the worst. Generally, the models could be divided into three groups with increasing accuracy: mel spectrogram, STFT, and MFCC. The significant difference in accuracy between the MFCC and STFT models compared to that of the mel-spectrogram model highlights the importance of feature extraction method selection.

### 4.3. Comparative Analysis of Classification Models

**Figure 12:**
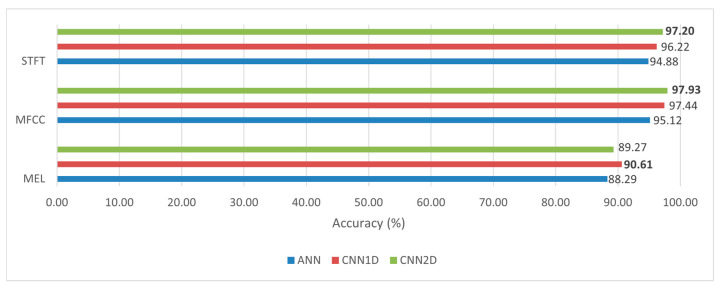
Comparative analysis of accuracy of different classification models. Remarks: bold number represent the highest accuracy achieved.

Based on the comparison depicted in Figure 12, the 2D CNN model demonstrated superiority over the 1D CNN and ANN models when utilizing the MFCC and STFT features. However, when employing the mel-spectrogram feature, the 1D CNN model showed a slight advantage over the 2D CNN model. The utilization of Doppler radar data in the form of a two-dimensional matrix enabled the 2D CNN classifier to achieve significantly enhanced prediction accuracy compared to that of the 1D CNN and ANN models.

**Figure 13:**
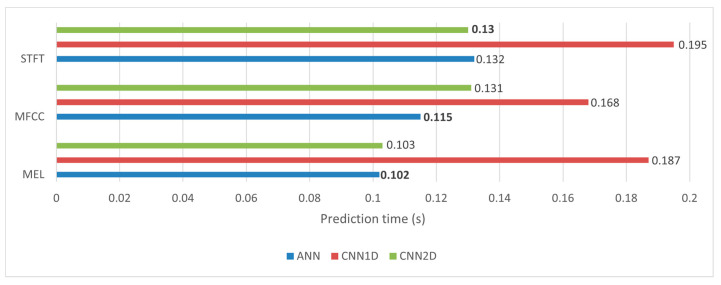
Comparative analysis of prediction time of different models. Remarks: bold number represent the highest accuracy achieved.

When evaluating classification models, it is important to consider parameters such as prediction time and model size in addition to classification performance. As illustrated in Figure 13, the ensemble of 1D CNN models exhibited a notably longer prediction time, spanning from 0.168 to 0.195 s, in comparison to that of the other models. The ANN model group had a relatively shorter prediction time (around 0.102 to 0.132 s). The 2D CNN model group had an acceptable prediction time, with a time difference of about half that of the 1D CNN model group compared to the ANN model group. In terms of model size, both the proposed 2D CNN model and the 1D CNN model showcased a relatively compact size of approximately 13 MB. In contrast, the ANN model presented a slightly larger model size of approximately 16.5 MB. While these models are not considered extremely lightweight, they still remain highly competitive when compared to other popular models [37], such as AlexNet (23.8 MB), GoogleNet (40 MB), and ResNet-50 (100 MB).

**Figure 14:**
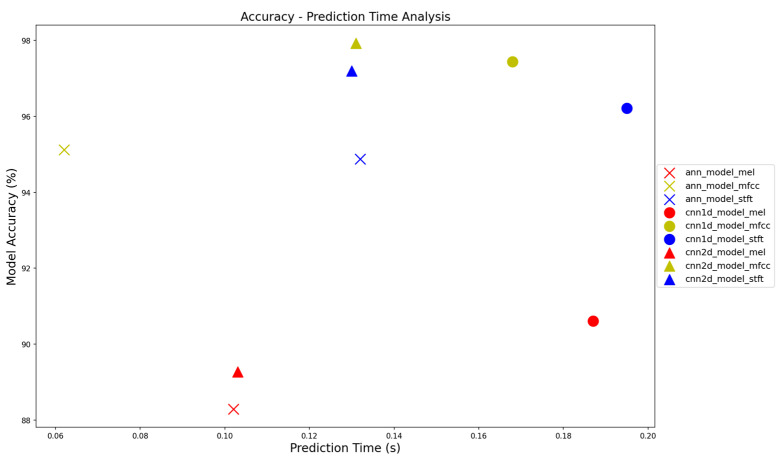
The graph provides a summary and comparison of the classification performance and prediction time of the models trained on various features.

Three classification models (ANN, CNN 1D, and CNN 2D) were trained on three different features, resulting in nine models, all of which were tested on the dataset. The analysis of the results included accuracy, recall index, F1-score, and the AUC (Table 2). The most accurate model was the 2D CNN trained on the MFCC feature with the highest F1-score of 98.05%. To establish a general relationship between accuracy and prediction time, a distribution chart was generated and is presented in Figure 14. The data points corresponding to the MFCC feature are clustered in the upper-left quadrant of the chart, indicating a high classification rate and acceptable prediction time. In contrast, the mel-spectrogram feature yielded the worst performance, with its data points distributed in the bottom area of the chart.

Based on the results presented in Table 3, our method achieved a remarkable accuracy of up to 97.73%, which surpasses that of most previous studies utilizing different data acquisition and classification techniques. The candidates for comparison cover a broad range of both classification and representation methods. These classification models range from traditional machine-learning techniques like SVM to more modern approaches such as XGBoost. Moreover, these studies also encompass a variety of use cases and signal-processing methods.

## 5. Conclusions

A 24 GHz Doppler radar data acquisition system was successfully developed, integrating both hardware and software components. It generated a labeled Doppler radar dataset for three target subclasses: non-human motion, human walking with arm swing, and human walking without arm swing, utilizing the designed hardware system. Ease of use was a primary focus during the design process, resulting in a user-friendly “plug-and-play” system that can be easily adopted if the design is followed. This system consistently captures highly reliable data, as demonstrated by the accurate performance of the object classification models trained on micro-Doppler features.

The project implemented advanced processing algorithms and utilized traditional artificial intelligence (AI) models for classification based on micro-Doppler features associated with human walking motion. The results indicated that these specialized processing techniques, mel spectrogram and MFCC, which are widely adopted in the domain of speech and audio, have demonstrated superior effectiveness in analyzing Doppler signals, especially MFCC, which surpassed the traditional STFT transformations. With the utilization of three different models for classification, namely artificial neural networks (ANN), 1D convolutional neural networks (CNN), and 2D CNN, the 2D CNN model trained on the MFCC feature achieved the highest accuracy of 97.93% for human motion micro-Doppler radar signals. Moreover, these models exhibited compact sizes and fast prediction times, with the 2D CNN model trained on the MFCC feature achieving a remarkable prediction time of 0.14 s.

However, it is important to consider the potential limitations of the study. One such limitation is the relatively small sample size, consisting of only six participants. While the data obtained from this sample has provided valuable insights, it may not fully capture the diversity and complexity of authentication scenarios in real-world applications. Thus, it is recommended that future investigations involve a larger participant pool to ensure greater representativeness and generalizability. Expanding the range of motions beyond walking would enhance the applicability of the findings. Incorporating additional activities and movements can provide a more comprehensive understanding of the system’s performance and capabilities.

Although this study proves MFCCs to be an effective method for processing Doppler radar signal specifically for human motion, exploring more sophisticated models for classification could be an interesting avenue for further improvement. By leveraging advanced algorithms and techniques, it is possible to enhance both the prediction time and model size, leading to more efficient and effective results.

## Figures and Tables

**Table 1:** Values of classifier training parameters.

Training Parameters	Value
Training set	72%
Validation set	8%
Test set	20%
Random shuffle	Yes
Number of Epoch	200
Batch size	32
Initial learning rate	1 × 10^−4^
Optimizer	Adam

**Table 2:** Summary table of results of accuracy indicators, F1-score, recall, and average AUC.

No.	Method	Accuracy	Precision	F1-Score	Recall	AUC
1	ann_model_mel	88.29	0.8903	0.8903	0.8904	0.9429
2	cnn2d_model_mel	89.27	0.9006	0.8991	0.8987	0.9567
3	cnn1d_model_mel	90.61	0.9129	0.9121	0.9128	0.9566
4	ann_model_stft	94.88	0.9514	0.9516	0.9519	0.9884
5	ann_model_mfcc	95.12	0.9598	0.9539	0.9526	0.9971
6	cnn1d_model_stft	96.22	0.9650	0.9641	0.9634	0.9931
7	cnn2d_model_stft	97.20	0.9750	0.9736	0.9731	0.9964
8	cnn1d_model_mfcc	97.44	0.9760	0.9760	0.9764	0.9979
9	cnn2d_model_mfcc	97.93	0.9807	0.9805	0.9802	0.9979

**Table 3:** Comparison of accuracy with other methods that use different data acquisition and classification approaches.

	Classification Method	Signal Representation Method	Accuracy	Categories
[8]	Bagged Trees	S-method	97.30%	Walking motions
[38]	SVM	STFT	94.00%	Human activities
[21]	CNN	CEMD	96.32%	Hand sign language
[39]	DivNet	STFT	97.00%	Human activities
[40]	Hidden Markov	MFCC	97.00%	UAV detection
[41]	XGBoost	MFCC	87.38%	Breathing pattern
Proposed system	2DCNN	MFCC	97.93%	Walking motions

## Data Availability

Raw Doppler data were collected at the University of Science, VNU-HCM, Vietnam. The derived data supporting the findings of this study are available from the authors on request.
